# The roles of FOXM1 in pancreatic stem cells and carcinogenesis

**DOI:** 10.1186/1476-4598-12-159

**Published:** 2013-12-10

**Authors:** Ming Quan, Peipei Wang, Jiujie Cui, Yong Gao, Keping Xie

**Affiliations:** 1 Department of Oncology and Shanghai Key Laboratory of Pancreatic Diseases, First People’s Hospital, School of Medicine, Shanghai Jiaotong University, Shanghai, People’s Republic of China; 2 Department of Oncology and Tumor Institute, Shanghai East Hospital, Tongji University School of Medicine, Shanghai, People’s Republic of China; 3 Department of Gastroenterology, Hepatology & Nutrition, The University of Texas MD Anderson Cancer Center, 1515 Holcombe Boulevard, Houston, TX 77030, USA

**Keywords:** Transcription factors, Oncogenic switch, Progression, Stem cells, Therapeutic targets, Molecular biomarkers

## Abstract

Pancreatic ductal adenocarcinoma (PDAC) has one of the poorest prognoses among all cancers. Over the past several decades, investigators have made great advances in the research of PDAC pathogenesis. Importantly, identification of pancreatic cancer stem cells (PCSCs) in pancreatic cancer cases has increased our understanding of PDAC biology and therapy. PCSCs are responsible for pancreatic tumorigenesis and tumor progression via a number of mechanisms, including extensive proliferation, self-renewal, high tumorigenic ability, high propensity for invasiveness and metastasis, and resistance to conventional treatment. Furthermore, emerging evidence suggests that PCSCs are involved in the malignant transformation of pancreatic intraepithelial neoplasia. The molecular mechanisms that control PCSCs are related to alterations of various signaling pathways, for instance, Hedgehog, Notch, Wnt, B-cell-specific Moloney murine leukemia virus insertion site 1, phosphoinositide 3-kinase/AKT, and Nodal/Activin. Also, authors have reported that the proliferation-specific transcriptional factor Forkhead box protein M1 is involved in PCSC self-renewal and proliferation. In this review, we describe the current knowledge about the signaling pathways related to PCSCs and the early stages of PDAC development, highlighting the pivotal roles of Forkhead box protein M1 in PCSCs and their impacts on the development and progression of pancreatic intraepithelial neoplasia.

## Introduction

The incidence of pancreatic cancer is increasing annually, especially in industrialized countries [1]. Despite ever-increasing research efforts over the past few decades, prognoses for pancreatic cancer remain among the poorest for all cancers. It is also one of the leading causes of cancer-related mortality in developed countries, with a median survival duration of 6 months and 5-year overall survival rate of less than 5% [2,3]. Conventional therapies, such as surgery, radiation therapy, chemotherapy, and combinations of them, have had a limited impact on the course of this aggressive neoplasm, which is characterized by rapid metastasis and resistance to these therapies [4]. Researchers recently demonstrated that the presence of cancer stem cells (CSCs) in pancreatic tumors contributes to the early metastasis and chemotherapeutic drug resistance of pancreatic cancer [5]. Therefore, elucidating the molecular mechanisms underlying the critical roles of pancreatic CSCs (PCSCs) in pancreatic cancer development and progression is imperative.

CSC research has resulted in many advances in the fundamental understanding and clinical management of several solid tumors, including brain, breast, head and neck, lung, prostate, colon, ovarian, and pancreatic cancer [5-11]. CSCs are now widely accepted to be a subpopulation of tumor cells with the capacity for extensive proliferation, self-renewal, multipotency, high tumorigenicity, and treatment resistance. Moreover, CSCs have a high propensity for invasiveness and metastasis [12]. CSCs in pancreatic cancer cases are characterized by expression of the cell surface markers CD44, CD24, and epithelial-specific antigen (ESA; epithelial cell adhesion molecule [EpCAM]) [13]. Authors reported that CD133^+^ cells in primary pancreatic tumors and pancreatic cancer cell lines represent those with enhanced, potent proliferative capacity [14]. Increasingly, studies have demonstrated that the presence of PCSCs combined with drug resistance and high levels of metastasis contribute to therapy failure, resulting in the high mortality rates for pancreatic cancer [5]. Furthermore, researchers have proposed that Forkhead box protein M1 (FOXM1) is involved in the self-renewal of PCSCs, and tumorigenesis and metastasis of pancreatic cancer cells [15].

FOXM1 is a member of the Forkhead box transcription factor superfamily, which consists of more than 50 members sharing a conserved winged-helix DNA-binding domain. FOXM1 is a proliferation-specific transcription factor whose expression is correlated with the proliferative ability of cells [16]. FOXM1 is well known to be a key cell-cycle regulator for both transition from G1 to S phase and progression from G2 phase to mitosis [17]. Increasing evidence suggests that FOXM1 expression is substantially elevated in most human malignancies, such as glioblastoma, lung cancer, hepatocellular carcinoma (HCC), breast cancer, and pancreatic cancer, and plays a crucial role in tumorigenesis, angiogenesis, invasion, and metastasis [17-23]. Also, several recent studies suggested that FOXM1 is involved in self-renewal and proliferation of CSCs [15,24,25]. However, the molecular mechanisms by which FOXM1 signaling regulates PCSCs in pancreatic cancer development and progression remain poorly understood.

A deeper comprehension of PSCSs would likely provide a new perspective on and increased understanding of the mechanisms that govern the development of pancreatic cancer. In this review, we briefly describe the crucial role of FOXM1 in PCSCs in pancreatic cancer development and progression with a focus on recent insight into the cross-talk between FOXM1 and signaling pathways in PCSCs here and below.

## The roles of FOXM1 and signaling pathways in the early stages of pancreatic ductal adenocarcinoma development

**Figure 1:**
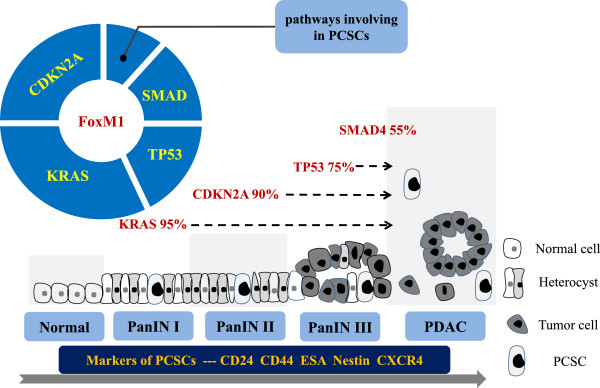
**Model of the genetic progression of pancreatic carcinogenesis.** The genetic alterations that occur during pancreatic carcinogenesis can be classified as early (activating mutation of KRAS), intermediate (inactivation of CDKN2A), and late (inactivation of TP53 and SMAD4 and activation of some pathways in PCSCs) events. Markers of PCSCs, including CD24, CD44, CXCR4, ESA, and Nestin, are detected in different sites during pancreatic carcinogenesis (in order of increasing percentage): normal ducts, low-grade PanIN lesions, high-grade PanIN lesions, and PDACs. FOXM1 may play a critical role in the early stages of PDAC development via cross-talk with major signaling pathways. Other gene mutations may occur during PanIN formation but are not illustrated in this model.

Over the past few decades, increasing evidence has demonstrated that almost all pancreatic cancers progress from diverse premalignant lesions to invasive carcinomas. Precursors of pancreatic cancer include pancreatic intraepithelial neoplasia (PanIN), intraductal papillary mucinous neoplasms (IPMNs), mucinous cystic neoplasms (MCNs), and intraductal tubular papillary neoplasms (ITPNs) [26-28]. Pancreatic carcinoma in general may arise from any of these precursor lesions, yet pancreatic ductal adenocarcinoma (PDAC) in particular is much more closely associated with PanIN than with the other precursor lesions. PanIN lesions are classified as PanIN-1, -2, or -3 based on the degree of morphologic atypia and the genetic events during pancreatic carcinogenesis [26,28]. In this section, we describe the roles of signaling pathways related to PanIN in the early stages of PDAC development and the cross-talk between FOXM1 and these pathways (Figure 1).

A comprehensive genetic analysis of 24 pancreatic cancer cases identified a total of 1562 somatic mutations. Further categorization of these alterations using whole exome sequencing revealed that they corresponded to 12 core signaling pathways, which contained several important genes already confirmed to be drivers in the early stages of PDAC development, such as cellular processes (KRAS), DNA damage control (tumor suppressor 53 [TP53]), cell-cycle regulation (cyclin-dependent kinase inhibitor 2a [CDKN2A]), and transforming growth factor (TGF)-β signaling (SMAD4) [29]. The progression of PanIN from trivial dysplastic epithelium (PanIN-1) to cytologic and nuclear atypia (PanIN-2 and −3, respectively) and, ultimately, invasive carcinoma corresponds to the gradual accumulation of genetic alterations. These alterations include activating mutation of the oncogene KRAS and inactivation of the tumor suppressor genes CDKN2A, TP53, and SMAD4 [28,30,31].

### KRAS

The earliest and most important genetic alterations observed in pancreatic carcinogenesis are activating mutations of KRAS [32-34]. At least 90% of PanIN-1 lesions harbor mutations of KRAS, and the average concentration of mutant KRAS alleles in PanIN lesions increases markedly along with the PanIN grade; ultimately, almost all PanIN lesions have KRAS mutations [30,35]. Researchers have identified three major point mutations in codons 12, 13, and 61 that lead to the association of abnormal KRAS protein products with malignant transformation, with mutations in codon 12 being the most important to pancreatic carcinogenesis [32,33]. KRAS encodes for a guanosine triphosphate (GTP)-binding protein that functions as a crucial modulator of a series of cellular processes, such as proliferation, survival, and motility. Ras activity in cells is tightly regulated, and Ras is normally bound to guanosine diphosphate (GDP) in an inactive state. Exogenous signals such as growth factor stimulation trigger the removal of GDP from Ras, allowing for GTP to bind and activate downstream effectors. Activated ras feeds multiple signaling pathways, including the Ras/Raf/mitogen-activated protein kinase (MAPK) pathway and the phosphoinositide 3-kinase (PI3K)/AKT signaling pathway, which have diverse roles in cytoskeletal alterations, cell-cycle progression, and apoptosis inhibition. Activating mutations of KRAS contribute to loss of intrinsic GTPase activity of Ras protein; consequently, continuous activation of ras occurs even in the absence of extracellular signals [34]. To determine the role of oncogenic RAS mutations in PanIN development and progression, Hingorani and colleagues directed endogenous expression of KRAS^G12D^ to progenitor cells in the mouse pancreas and found that physiologic levels of KRAS^G12D^ activity induced the formation of ductal lesions that recapitulate the full spectrum of PanIN lesions, which are highly proliferative and histologically progressive, and exhibit various activated signaling pathways that are quiescent in normal ductal epithelium [36,37]. Almost all PanIN lesions and invasive pancreatic carcinomas harbor oncogenic mutations of KRAS, furthermore, a small fraction of KRAS-wild-type PDACs have BRAF mutations, similarly resulting in aberrant MAPK signaling [38]. These results indicated that KRAS activation is an important initiating step in the early stages of PDAC development and that this activation leads to the onset of most cases of pancreatic tumorigenesis.

### CDKN2A (p16)

Researchers have observed inactivation of the p16 gene, also known as CDKN2A as well as multiple tumor suppressor 1, by deletion, point mutation, or methylation of the promoter region of the gene in more than 90% of PDAC cases, with the resultant loss of activity of p16 protein, a key regulator of the transition from G1 to S phase in the cell cycle, corresponding to increased cell proliferation [39-41]. The vast majority of inactivation of CDKN2A arises as early as the PanIN-2 stage [39,40]. P16 primarily functions in cell-cycle control as a negative regulator of the extraordinary pRb/E2F signaling pathway. At the G1-S transition, p16 specifically inhibits CDK4/6-mediated phosphorylation of Rb, the retinoblastoma-susceptible gene product, thus sequestering the transcription factor E2F as incompetent pRb/E2F complexes and inhibiting cell-cycle progression [42]. In addition, the CDKN2A gene alternative splicing sites that lead to formation of several protein products, such as p14, may sequester MDM2, helping stabilize TP53 [43].

### TP53

TP53 is a homotetrameric complex that transactivates key target genes in response to a series of cellular processes, including cell-cycle progression, apoptosis, and DNA damage response. DNA damage specifically activates p53 protein, which promotes DNA damage repair or leads to cell-cycle arrest at G1 phase and induces apoptosis [43]. About 75% of pancreatic cancer cases have TP53 inactivation, including that caused by gene mutation and/or abnormal nuclear accumulation of p53 protein, with the vast majority of genetic alterations occurring in PanIN-3 lesions [44-46]. Hingorani et al. [47] targeted concomitant endogenous expression of Trp53^R172H^ (a TP53 mutant) and Kras^G12D^ to the mouse pancreas, resulting in cooperative promotion of chromosomal instability and development of highly metastatic PDAC from early-stage, preinvasive lesions with Kras^G12D^ expression.

### SMAD4

The Smad4 gene, also known as tumor suppressor DPC4 (deleted in pancreatic cancer cells), is inactivated in about 55% of PDAC cases, either by homozygous deletion or intragenic mutation with loss of its second allele [48]. As with TP53, investigators have observed loss of Smad4 expression in PanIN-3 lesions [49]. The Smad4 protein has a crucial role in propagation of extracellular signaling pathways via the TGF-β signaling pathway, which modulates cell proliferation and differentiation, thus functioning as a critical tumor suppressor in normal cells. This signaling pathway is activated when TGF-β binds to type I and II serine/threonine kinase cell surface receptors, resulting in receptor dimerization and subsequent phosphorylation of receptor I by receptor II. Activation of receptor I leads to phosphorylation of Smad2 and Smad3 proteins, with which Smad4 forms complexes, thus corporately translocate into the nucleus. Once in the nucleus, these complexes can associate with transcriptional co-factors and regulate expression of the target genes involved in a series of crucial cellular processes [50]. Deletion of Smad4 in mice on a mutant Kras (Kras^G12D^) background resulted in faster formation of PanIN lesions and greater fibrosis, with some mice having invasive tumors, than did the Kras^G12D^ background alone. However, mice with Smad4 deletion alone had no obvious pathological changes [51]. Further research of the role of Smad4 in pancreatic carcinogenesis demonstrated that concomitant expression of Kras^G12D^ and haploinsufficiency of the Smad4 tumor suppressor gene resulted in development of invasive PDAC from mucinous cystic neoplasms (MCNs) in a mouse model [52]. Loss of Smad4 expression correlates with both development of widespread metastasis and poor prognosis in pancreatic cancer patients [53,54].

## Signaling pathways in PCSCS

Carcinoma and stem cells have many of the same properties, such as sustained proliferative capacity, immortality, and self-renewal, all of which are associated with carcinogenesis. Evidence supporting the viewpoint that pancreatic tumors contain a distinct subpopulation of self-renewing tumor cells -CSCs that are responsible for tumorigenesis and metastasis in PDAC cases continues to mount [55]. Researchers have identified several CSC-specific markers, including CD24, CD44, CD133, ESA, Nestin, and combinations of them [13,14,56]. In a recent study, investigators detected CD24-, CD44-, CXCR4-, ESA-, and Nestin-positive cells in the following tissues (in order of increasing percentage): normal ducts, low-grade PanIN, high-grade PanIN, and PDAC tumors. This suggested that most CSC markers correlate with pancreatic tumorigenesis in the PanIN-to-PDAC sequence of progression [57]. Another group of researchers found that the homeobox transcription factors Oct4 and Nanog (stem cell-specific transcription factors) were overexpressed in metaplastic ducts and that Oct4 expression preceded Kras mutation, which indicated that these CSC-specific transcription factors are associated with early stages of pancreatic carcinogenesis and may play important roles in that process [58]. In addition, some aberrant signaling pathways in PCSCs, such as Notch [59-61], PI3K/AKT/phosphatase and tensin homolog deleted from chromosome 10 (PTEN) [62], B-cell-specific Moloney murine leukemia virus insertion site 1 (Bmi1) [63], c-Myc [64], and c-Met [65], participate in pancreatic carcinogenesis via stimulation of oncogenic Kras-dependent malignant transformation of PanIN.

## Crosstalk between FOXM1 and the signaling pathways in PCSCS

The transcription factor FOXM1 is a regulator of a wide spectrum of biologic processes in tumors, including cell-cycle progression, cellular proliferation, cellular differentiation, DNA damage repair, apoptosis, tissue homeostasis, and angiogenesis. Several studies have demonstrated that the FOXM1 signaling network is frequently deregulated in human malignancies with leading to its overexpression, which is associated with poor prognosis for various cancers, including pancreatic cancer [22,66]. These findings point to a principal role for FOXM1 in the pathogenesis and progression of pancreatic cancer via its involvement in progression, proliferation, angiogenesis, epithelial-to-mesenchymal transition (EMT), invasion, and metastasis [67-69].

Increasing evidence suggests that pancreatic carcinogenesis is a stepwise progression from epithelial precursor lesions to invasive PDAC via successive genetic alterations, including activation of the oncogene Kras and inactivation of the tumor suppressor genes CDKN2A, TP53, and Smad4. Furthermore, some signaling pathways in CSCs play a role in this progression. Abnormal activation of Kras in the Ras/Raf/MAPK and PI3K/AKT pathways plays a pivotal role in cell-cycle progression and apoptosis inhibition. FOXM1 has close relationships with both of these signaling pathways. Specifically, Ras/Raf/MAPK stimulates the nuclear translocation and transactivating activity of FOXM1 [70], and upregulation of FOXM1 expression is mechanistically linked with hyperactivation of the PI3K/AKT pathway and loss of function of TP53 [71]. Also, investigators found that constitutive expression of FOXM1 cooperated with activated Kras to induce lung cancer growth *in vivo*[72]. A recent study demonstrated that upregulation of FOXM1 expression suppressed the expression of CDKN2A via promoter hypermethylation [73]. The results of another study demonstrated that aberrant upregulation of FOXM1 expression induces genomic instability, which abolishes the normal checkpoint response to DNA damage (e.g., p53, p16). Consequently, damaged cells are allowed to proliferate, and the genetic aberrations or mutations required for tumor initiation can take place [74]. A study of malignant neuroblastoma suggested that FOXM1 plays a pivotal role in the tumorigenicity of these aggressive tumor cells via maintenance of their self-renewal capacity [15]. Additional studies demonstrated that FOXM1 plays a key role in maintenance of stem cell pluripotency *in vivo* by inducing the expression of pluripotency genes, including Oct4, Nanog, and Sox2 [75]. Taken together, these findings provide convincing evidence that FOXM1 plays a central role in the early stages of PDAC development via cross-talk with signaling pathways related to PanIN and PSCSs.

## Major signaling pathways in PCSCS

**Figure 2:**
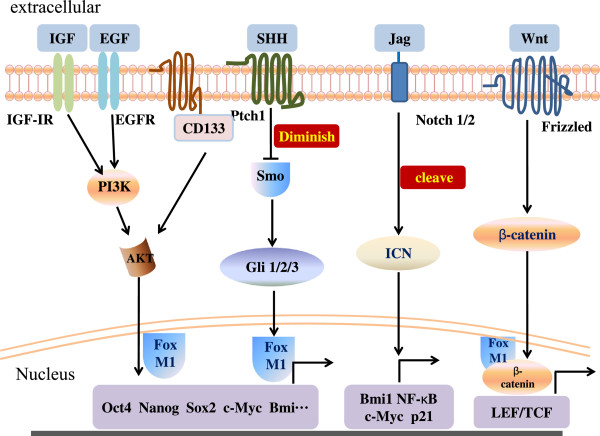
**Signaling pathways in PCSCs.** The HH and Notch developmental pathways are highly active in PCSCs and may be activated by a series of respective ligands. The SHH/Gli signaling pathway plays a pivotal role in maintenance of stemness (self-renewal) via regulation of the expression of the pluripotency-maintaining factors Nanog, Oct4, c-Myc, and Sox2. Upon activation by interaction with ligands, Notch is cleaved and translocated to the nucleus for transcriptional activation of Notch target genes, including hairy and enhancer of split-1, nuclear factor κB (NF-κB), cyclin D1, and c-Myc. The Wnt/β-catenin pathway is vitally involved in cell fate determination via binding to the transcription factor T-cell factor/lymphocyte enhancer factor (TCF/LEF). PI3K/AKT signaling is involved in PCSCs by directly interacting with CD133. FOXM1 plays a pivotal role in PCSCs by directly stimulating stem-like characteristics and cross-talk with other pathways. Abnormal signaling pathways also may involve PCSCs but are not illustrated in this figure. IGF, insulin-like growth factor; EGF, epidermal growth factor; Jag, Jagged; IGF-1R, insulin-like growth factor-1 receptor; EGFR, epidermal growth factor receptor; ICN, intracellular domain of Notch.

Increasing evidence supports the existence of CSCs in pancreatic tumors. PCSCs make up a subpopulation of cells distinguishable from the majority of regular tumor cells because of their exclusive ability to drive tumorigenesis, invasion, metastasis, drug resistance, and disease relapse via extensive proliferation, self-renewal, and multipotency. Similar to common cancer cells, multiple abnormal signaling pathways are found in PCSCs, such as Hedgehog (HH), Notch, Wnt, Bmi, PI3K/AKT/PTEN, FOXM1, and Nodal/Activin [68,76-81]. In addition, the CSC niche is essential to the development of PCSCs (Figure 2) [82,83].

### HH Signaling

The HH signaling pathway is essential to embryonic pancreatic development and differentiation, and researchers have implicated the deregulation of this pathway in several forms of carcinomas [84]. Mounting evidence indicates that the HH signaling pathway is aberrantly activated and one of the majority mediators in PDAC cases [85]. Sonic HH (SHH) is the most important homologous gene in the HH family, which also includes Desert HH and India HH. Canonical signaling of this pathway is modulated by the transmembrane receptor Patched, which normally has an inhibitory effect on another transmembrane receptor, Smoothened (Smo). Upon binding with short-acting polypeptide ligands such as SHH, the Smo-suppressive function of Patched is diminished, thus allowing transduction via the SHH pathway, which brings about activation and nuclear translocation of the glioma-associated oncogene (Gli) family of zinc-finger transcription factors (Gli1, Gli2, and Gli3). Ultimately, these factors activate transcription of SHH target genes involved in cellular proliferation, progression, survival, and stemness and cell-fate determination, such as FOXM1, Wnt, Bmi1, Nanog, Oct4, Sox2, Snail, Slug, and Bcl-2 [84,86]. Researchers have observed aberrant expression of SHH in PDAC tumors as well as PanIN lesions, suggesting that upregulation of SHH expression contributes to pancreatic cancer initiation, development, and progression [85]. In another study, inhibition of HH signaling greatly decreased cell proliferation and induced apoptosis via suppression of the PI3K/AKT pathway and markedly inhibited EMT by suppressing activation of the transcription factors Snail and Slug, whose expression is correlated with pancreatic cancer cell invasion, suggesting that the HH signaling pathway is involved in the early stages of metastasis [87].

Recently, multiple lines of evidences supported that the SHH/Gli signaling pathway is highly activated in PCSCs and plays a pivotal role in maintenance of stemness (self-renewal) by regulating the expression of pluripotency-maintaining factors, including Nanog, Oct4, c-Myc, and Sox2 [77,88-92]. Both sulforaphane and the combination of epigallocatechin-3 gallate and quercetin inhibit the self-renewal capacity of PCSCs via attenuation of the SHH/Gli pathway [88-91]. The Gli transcription factor inhibitor GANT-61 inhibits PCSC viability, spheroid formation, Gli-DNA binding, and transcriptional activity and induces apoptosis. Furthermore, GANT-61 inhibits PCSC-containing tumor growth, which is associated with upregulation of TRAIL-R1/DR4 and TRAIL-R2/DR5 expression and downregulation of Gli-1, Gli-2, Bcl-2, and ZEB1 expression in tumor samples obtained from nude mouse xenografts [77]. Huang et al. [92] observed that Panc-1 tumorspheres have stemness potential, in which the SHH pathway is active as indicated by expression of the HH components Smo, Gli1, and Gli2. They also observed that treatment with the SHH inhibitor cyclopamine could reverse resistance to gemcitabine owing to decreased expression of the ATP-binding cassette transporter ABCG2 in PANC-1 tumorspheres. PCSCs are thought to be responsible for tumor maintenance, progression, and resistance to chemotherapy and radiation therapy. Recent studies demonstrated that the SHH/Gli pathway plays pivotal roles in chemoresistance caused by PCSCs based on ATP-binding cassette transporter overexpression [93]. Combined blockade of SHH and mammalian target of rapamycin (mTOR) signaling together with standard chemotherapy is capable of eliminating PCSCs [94]. Another study demonstrated that the combination of HH signaling inhibition and radiation therapy had more than additive effects on pancreatic tumorsphere regeneration *in vitro*[76]. Vismodegib (GDC-0449), an oral antagonist of the SHH signaling pathway, inhibits PCSC characteristics by blocking the activity of Smo *in vitro*[95]. A phase 1 trial of GDC-0449 is under way, preliminary results of which suggest that it has an acceptable safety profile and encouraging antitumor activity for some locally advanced or metastatic solid tumors [96]. Taken together, these findings deepen support the concept that the SHH signaling pathway is a fundamental driver of PCSCs.

### Notch signaling

The Notch signaling pathway is well known to be responsible for maintaining the balance between cell proliferation and death and plays instrumental roles in the formation of multiple human tumors, including pancreatic cancer [60,97]. Notch genes encode for proteins that can be activated via interaction with a family of their ligands. Humans have four Notch receptors—Notch1, Notch2, Notch3, and Notch4—with five related ligands—Delta-like1, Delta-like3, Delta-like 4, Jagged1, and Jagged2. Upon activation, Notch is cleaved, releasing the intracellular domain of Notch, which then can be translocated to the nucleus for transcriptional activation of Notch target genes, including hairy and enhancer of split-1, nuclear factor κB, cyclin D1, and c-myc [98]. Emerging evidence clearly suggests that activation of the Notch signaling pathway is mechanistically associated with molecular characteristics of CSCs in PDAC cases [79,99]. For example, Forced overexpression of Notch1 may increase the formation of pancreatospheres, which is consistent with expression of the CSC surface markers CD44 and EpCAM. This suggests that activation of Notch1 signaling is related to the self-renewal capacity of PCSCs [78]. Authors recently reported that expression of Oct4, Nanog, and PDX1 as markers of self-renewal of PCSCs occurred in Notch2^+^ BxPC-3 and Panc-1 human pancreatic cancer cells [100]. Also, these investigators found that expression of ALDH, a PCSC surface marker, was associated with poor overall survival durations in PDAC patients. Treatment with PF-03084014, a selective γ-secretase inhibitor, alone or in combination with gemcitabine is effective in reducing the number of ALDH^+^ tumor cells [101]. In a glioblastoma study, Notch blockade by GBI appeared to deplete stem-like cancer cells via reduced proliferation and increased apoptosis associated with decreased AKT and signal transducer and activator of transcription 3 phosphorylation, which was consistent with reduced expression of the CSC markers CD133, Nestin, Bmi1, and olig2 [102]. A growing body of published reports strongly suggest that Notch signaling is biologically relevant to CSCs in pancreatic cancer.

## BMI1, WNT, and other signaling pathways

Expression of Bmi1, a member of the polycomb group of transcriptional repressors, is markedly upregulated in pancreatic cancer cell lines and resected pancreatic tumor specimens, and this protein is related to proliferation of pancreatic cancer cells and survival, and prognosis in pancreatic cancer patients [103]. Emerging evidence demonstrates that Bmi1 plays a key role in the function of CSCs in PDAC cases [80,104]. Yin et al. [104] discovered that Bmi1 was more highly expressed in PANC-1 CSCs than in regular PANC-1 cells, with overexpression of cyclin D1 and ABCG2 and downregulation of expression of p16. A more recent study demonstrated that PCSCs had much higher expression of Bmi1 mRNA than did normal pancreatic tissue cells and marker-negative bulk pancreatic tumor cells. Bmi1 silencing in PCSCs inhibited secondary and tertiary tumorsphere formation, decreased primary pancreatic tumor xenograft growth, and decreased the proportion of CSCs in the xenografts [80]. These results demonstrated a key role for Bmi1 in maintenance of the PCSC compartment by regulating the cells’ self-renewal.

PI3K/AKT signaling plays a vital role in many biologic processes in PDAC cases, including cellular proliferation, differentiation, and survival. Recent studies demonstrated that PI3K/AKT signaling is involved in CSCs in diverse types of cancer, including pancreatic cancer [94,105,106]. Based on the results of studies of glioblastoma, PTEN, a regulator of PI3K/AKT signaling, appears to function as a crucial inhibitor of proliferation of CSCs and as an inducer of senescence, suggesting that the PTEN/PI3K/AKT axis is a fundamental signaling pathway in glioblastoma stem cells [105]. Researchers also found that CD133, a crucial trigger of self-renewal and tumorigenesis of CSCs, interacted directly with the PI3K 85-kDa regulatory subunit, resulting in preferential activation of the PI3K/AKT pathway in glioblastoma stem cells [106]. Mueller et al. [94] recently revealed that CD133^+^ PDACs had particularly high levels of mTOR signaling, suggesting that the PI3K/AKT pathway also plays a dominant role in PCSCs. Single-agent therapy with the mTOR inhibitor rapamycin profoundly reduced the number of CD133^+^ PCSCs among pancreatic cancer cells. mTOR, which belongs to the PI3K superfamily, is the target of a complex signal transduction pathway. Overall, PI3K/AKT signaling is known to be deeply involved in PCSCs. However, further study is needed to determine the molecular mechanism underlying PCSCs regulation by PI3K/AKT signaling.

Wnt signaling is one of the most well researched molecular pathways that regulate CSC self-renewal and proliferation in patients with various cancers, including colorectal cancer and glioma [24,107]. However, evidence demonstrating the functions of Wnt signaling in PCSCs is lacking in the literature. The Wnt/β-catenin pathway, the canonical pathway of Wnt signaling, is vital to cell-fate determination via binding to the transcription factor, T-cell factor/lymphocyte enhancer factor, and subsequent transcription of Wnt target genes. A study of colon cancer implicated that a high level of Wnt/β-catenin signaling activity is one of the mechanisms that drive the transition from colitis to cancer by sustaining the tumor-initiating potential of colon CSCs [107]. In addition, Zhang et al. [24] reported that FOXM1, as a downstream component of Wnt signaling, controlled the self-renewal of glioblastoma-initiating cells (GICs) via interaction with β-catenin. These results indicated that deregulation of Wnt signaling may play a key role in PCSCs and that the specific mechanisms must be elucidated.

The embryonic morphogens Nodal and Activin belong to the TGF-β superfamily and are crucial regulators of embryonic stem cell fate via binding to the Activin-like type I and II receptors (ALK4 and ALK7). Nodal and Activin are secreted proteins that are expressed during embryonic development and essential for maintaining the pluripotency of human embryonic stem cells. Recent evidence demonstrated that Nodal and Activin were barely detectable in highly differentiated pancreatic cancer cells but markedly overexpressed in PCSCs and stroma-derived pancreatic stellate cells (PSCs). Knockdown or pharmacologic inhibition of expression of ALK4 and ALK7 in PCSCs abrogated their self-renewal capacity and tumorigenicity *in vivo* and reversed the resistance of orthotopically engrafted PCSCs to treatment with gemcitabine [81]. The same research team later reported that Nodal-expressing PSCs are pancreatic tumor stroma components important to creation of a paracrine niche for PCSCs and that secretion of Nodal and Activin by PSCs promoted sphere formation *in vitro* and invasiveness of PCSCs in an ALK4-dependent manner [83]. These data implied that Nodal/Activin signaling, which is involved in the paracrine niche at the tumor-stroma interface, drives the self-renewal and tumorigenicity of PCSCs.

## Role of FOXM1 in PCSCS

Authors have well documented that FOXM1 plays an important role in the development and progression of PDAC and that FOXM1 overexpression is associated with poor prognosis and advanced clinicopathologic stages of PDAC [22]. Recent studies using human and mammalian models revealed that FOXM1 has a role in promotion of tumorigenesis by stimulating stem cell-like characteristics in pancreatic cancer cells, including self-renewal capacity [15,72,75]. Accordingly, a lung tumorigenesis study demonstrated that overexpression of FOXM1 promoted Clara cell hyperplasia and cooperated with activated K-Ras to induce lung cancer development *in vivo*[72]. In addition, mouse model studies demonstrated that FOXM1 is involved in maintenance of the carcinogenicity of neuroblastoma cells and the self-renewal capacity of mouse neural stem/progenitor cells via induction of expression of the pluripotency genes Sox-2 and Bmi1 [15]. A study of P19 embryonal carcinoma cells revealed that expression of FOXM1 is repressed during retinoic acid-induced differentiation at early stages and correlated with decreased expression of pluripotent stem cell markers and that expression of FOXM1 protein is downregulated before expression of Oct4 and Nanog decreases upon differentiation. Expression of Oct4 and Nanog is diminished by knockdown of expression of FOXM1, and the Oct4 promoter is regulated directly by FOXM1. In differentiated cells, such as retinoic acid-induced P19 cells and human newborn fibroblasts, overexpression of FOXM1 alone restarts the expression of the pluripotency-related transcription factors Oct4, Nanog, and Sox2. Taken together, these findings provide convincing evidence of critical involvement of FOXM1 in maintenance of stem cell pluripotency [75]. That acquisition of the EMT phenotype and induction of the CSC or a cancer stem-like cell phenotype are highly interrelated is common knowledge. Bao et al. [25] recently reported that FOXM1 is deeply involved in acquisition of the EMT and CSC phenotypes in pancreatic cancer cells. Forced overexpression of FOXM1 led to increased self-renewal capacity of AsPC-1 human pancreatic cancer cells, which was consistent with enhanced expression of CSC cell surface markers such as CD33 and EpCAM.

Although strong evidence that FOXM1 directly affects PCSCs has been limited until now, the close relationships of FOXM1 with HH, Notch, Bmi1, PI3K/AKT, Wnt, and other signaling pathway determine its promotive role in PCSCs.

## Cross talk between FOXM1 and the HH signaling pathway

In 2002, authors first reported that expression of the SHH target Gli1 in primary basal keratinocytes and other human cell lines caused a significant elevation of FOXM1 mRNA expression and transcriptional activity, indicating that FOXM1 is a downstream target gene of Gli1 [108]. Pignot et al. [109] confirmed this in a recent study of transitional cell carcinoma of the bladder. A colorectal cancer study demonstrated that SHH, Gli1, and FOXM1 mRNA expression levels were higher in colorectal adenocarcinomas than in adjacent normal colon tissue [110]. The researchers also found strong correlations between expression of SHH and FOXM1 and between expression of Gli and FOXM1 in colorectal cancer cells. Exogenous SHH expression increased proliferation of colon adenocarcinoma-derived cells (HT-29 and CaCo2) *in vitro* by inducing Gli1 and FOXM1 transcription. Another study demonstrated that FOXM1 overexpression in non-small cell lung cancer cells was remarkably correlated with Gli1 expression, indicating SHH signaling activation [111]. In human HCC cases, FOXM1 protein overexpression was highly associated with increased tumor grade and advanced tumor stage. Additionally, investigators observed a strong association between the expression of Gli2 and that of FOXM1 in HCC cells, which is consistent with the concept that in human HCC cases, the SHH signaling pathway is involved in differentiation and proliferation of tumor cells, in part via induction of nuclear accumulation of Gli2 and subsequent upregulation of expression of FOXM1 [112]. Taken together, these findings are convincing evidence of tight cross-talk between FOXM1 and the HH signaling pathway.

## Cross talk between FOXM1 and the PI3K/AKT signaling pathway

Emerging evidence demonstrates determinate specific cross-talk between FOXM1 and the PI3K/AKT pathway. For example, Upregulation of FOXM1 expression in anaplastic thyroid carcinoma cells is mechanistically linked with loss of function of p53 and hyperactivation of the PI3K/AKT signaling pathway [71]. Additionally, Park et al. [113] recently reported that in addition to the well-characterized function of FOXM1 in proliferation, deregulation of FOXM1b expression is a major driving force for multiple steps of tumor metastasis via activation of the AKT/Snail1 pathway and stimulation of expression of stathmin, lysyl oxidase, lysyl oxidase like-2, and several other genes involved in metastasis. In a prior study, the same research team found that the continuous presence of FOXM1 was required for survival of tumor cells expressing activated AKT (escaping premature senescence and apoptosis caused by oxidative stress), which was attributed to FOXM1’s critical roles in regulation of reactive oxygen species activity [114].

## Cross talk between FOXM1 and other signaling pathways

Downregulation of Notch1 expression leads to inhibition of cell growth and apoptosis induction, which is mechanistically linked with downregulation of AKT and FOXM1 expression, suggesting that AKT and FOXM1 are downstream targets of Notch1 signaling [115]. Bmi1 plays an integral role in enhancing pancreatic tumorigenicity and the function of CSCs in PDAC development and progression. A recent investigation demonstrated that Bmi1 is a downstream target of FOXM1; this was supported by dose-dependent induction of Bmi1 protein and mRNA expression by FOXM1 and the finding that depletion of FOXM1 by RNA interference decreased Bmi1 expression. Using Bmi1 promoter reporters with wild-type and mutated c-Myc binding sites and short hairpin RNAs targeting c-Myc, the researchers in that study further found that FOXM1 activated Bmi1 expression via c-Myc, the expression of which was recently reported to be regulated by FOXM1 [116]. In addition, investigators demonstrated that FOXM1 is a downstream component of the Wnt signaling pathway and critical for β-catenin nuclear location and transcriptional activation. A conceivable molecular mechanism was that Wnt3a increased the expression level and nuclear translocation of FOXM1, which bound directly to β-catenin and enhanced its activation. Furthermore, they discovered that interaction between FOXM1 and β-catenin plays a critical role in the self-renewal and differentiation of GICs, as knockdown of FOXM1 or β-catenin expression substantially decreased the size and number of primary and secondary spheres formation and reduced the efficiency of neural colony formation in GICs [24,117].

## Role of FOXM1 in the PCSC niche

Thus far, we have extensively described the mechanisms responsible for self-renewal and maintenance of the undifferentiated status of stem cells. The stemness of stem cells seems to be sustained by interactions with other cells, as most stem cells isolated from tissue cannot be maintained independently *in vitro*. Over the past few years, researchers have made increasingly explicit standpoint that the stem cell niche provides a microenvironment that is pivotal to protecting and perpetuating the self-renewal and undifferentiated state of stem cells [118]. This role of the stem cell niche also extends to the field of cancer biology. Like somatic stem cells, CSCs rely on a stem cell niche, dubbed the CSC niche, for self-renewal and differentiation. The CSC niches include the glioblastoma, colorectal, and hepatic CSC and PCSC niches [12,82,119,120].

## FOXM1 and the hypoxic microenvironment

PDAC is characterized by an excessive number of desmoplastic reactions and a hypoxic microenvironment, as the mesenchymal tissue at the primary tumor site consists of high-density fibrotic stroma that compresses the tumor vasculature, giving rise to intratumoral hypoperfusion. Poor blood perfusion leads to highly hypoxic conditions, which induces expression of hypoxia-inducible factor (HIF)-1α, thereby inducing the transcription of genes that regulate a variety of important cellular processes. The hypoxic and fibrogenic microenvironment of PDAC comprises not only tumor cells but also surrounding stromal cells, such as stellate, endothelial, and infiltrating immune cells. Researchers demonstrated that hypoxic conditions resulted in a large increase in the expression of the neural stem cell markers CD133 and Nestin as well as the stem cell markers Oct4 and Sox2 [121]. Hypoxia also induced the human embryonic stem cell transcriptional program, including the induced pluripotent stem cell inducers Oct4, Nanog, Sox2, KLF4, c-Myc, and microRNA302, in 11 cancer cell lines [122]. In PDAC cases, hypoxia induces tumor aggressiveness, which is associated with expansion of the CD133^+^ pancreatic cancer cell population in a predominantly HIF-1α–dependent manner [123]. Hypoxia, a common feature of the microenvironments of solid tumors, induces expression of FOXM1 in tumorigenic cells owing to direct binding of HIF-1 to the HIF-1–binding sites in the FOXM1 promoter. Investigators found that transcriptional upregulation of FOXM1 expression accelerated the growth of hypoxic cancer cells by decreasing nuclear expression levels of p21 and increasing expression of cyclin B1 and cyclin D1 [124]. As described above, low oxygen levels in the PCSC niche may be of great importance to the development of PCSCs in an HIF-1α–dependent manner, and FOXM1 may be involved in this process.

## FOXM1 and the TGF-β signaling pathway

Hypoxia exists around not only cancer cells but also surrounding PSCs, which are abundantly present in the stroma containing pancreatic cancer cells and may serve as a CSC niche. Hypoxia stimulates PSCs to induce fibrosis and angiogenesis in PDAC tumors by facilitating migration, type-I collagen expression, and vascular endothelial growth factor production in PSCs. Furthermore, the presence of stromal desmoplasia is a hallmark of PDAC, forming a unique microenvironment that comprises many cell types. PSCs have been identified to play a key role in pancreatic cancer desmoplasia. Authors reported that conditioned media of hypoxia-treated PSCs promoted endothelial cell proliferation and migration and angiogenesis *in vitro* and *in vivo* accompanied by expression of several angiogenesis-regulating molecules, including vascular endothelial growth factor receptor, angiopoietin-1, and Tie-2 [125]. A recent study demonstrated that the presence of PSCs enhanced the CSC-like phenotypes in pancreatic cancer cells. The results of this study indicated that indirect co-culture of pancreatic cancer cells and PSCs enhanced the spheroid-forming ability of the cancer cells and induced expression of the PCSC-related genes ABCG2 and Nestin [126]. Lonardo et al. [83] demonstrated that Nodal/Activin-expressing PSCs are major components of the pancreatic tumor stroma, providing a paracrine niche for PCSCs. Nodal and Activin are crucial regulators of embryonic stem cell fate. They are barely detectable in differentiated tumor cells but markedly overexpressed in PCSCs and PSCs [81]. Secretion of Nodal and Activin by PSCs promotes sphere formation *in vitro* and PCSC invasiveness *in vivo*[83]. These studies suggested that the Nodal/Activin pathway is essential to the self-renewal capacity and stemness properties of PCSCs [81,83].

In addition to Nodal and Activin, other TGF-β family members, together with other pathways, form a network that regulates the acquisition and/or maintenance of CSC properties via modulation of the tumor microenvironment [127,128]. TGF-β–induced EMT can guide cancer cells to dedifferentiate and gain CSC-like properties. Furthermore, EMT facilitates generation of stromal cells that serve as a niche for CSCs [127]. Researchers found that the side population of pancreatic cancer cells, a CSC-enriched fraction from a pancreatic cancer cell line, possessed great potential to switch the cells’ phenotype between mesenchymal and epithelial via TGF-β stimulation or elimination [128]. Additionally, autocrine TGF-β signaling is involved in the maintenance and survival of stem-like cell populations [129], and exposure of tumor cells to TGF-β and tumor necrosis factor-α induces EMT, which generates tumor cells with stem cell properties [130,131]. Expression of Bone morphogenetic protein, another TGF-β family member, is necessary for the self-renewal of embryonic stem cells via inhibition of differentiation [132]. Medici and colleagues discovered that TGF-β2 and bone morphogenetic protein 4 were stimulators of the conversion of vascular endothelial cells into multipotent stem-like cells [133]. As described above, TGF-β signaling, together with other reported and/or unknown pathways, may regulate the acquisition and/or maintenance of the stemness of PCSCs, creating a malignant stem cell niche.

Although little is known about the cross-talk between FOXM1 and PSCs, emerging evidence demonstrates the existence of cross-talk between FOXM1 and TGF-β pathways [134,135]. FOXM1 is involved in TGF-β1–induced EMT, and TGF-β–based treatment has led to a dramatic increase in FOXM1 expression in non-small cell lung cancer cells [134]. A recent study demonstrated that transgenic expression of activated FOXM1 in alveolar epithelial cells upregulated radiation-induced expression of EMT-associated genes, including interleukin-1β, Snail1, Snail2, Zeb1, Zeb2, Twist2, and Foxf1; reciprocally, conditional deletion of FOXM1 from respiratory epithelial cells prevented an increase in EMT-associated gene expression. Furthermore, a study demonstrated that FOXM1 induced EMT by binding to and increasing the promoter activity of the Snail gene, a crucial transcriptional regulator of EMT [135]. The investigators also found that FOXM1 expression was induced in alveolar epithelial cells after lung irradiation and that this induction strengthened radiation-induced pneumonitis and pulmonary fibrosis. In contrast, inhibition of FOXM1 expression diminished fibrosis [135].

## FOXM1 and tumor-associated macrophages

Tumor-infiltrating immune cells are a hallmark of most solid tumors, and accumulating evidence has demonstrated that the presence of varied immune populations significantly affects prognosis in various mouse and human malignancies [136,137]. Macrophages that infiltrate and interact with cancer cells, *i.e.*, tumor-associated macrophages (TAMs), are the dominant immune cell components and play indispensable roles in tumor development and progression through secreting numerous cytokines, chemokines and growth factors, which promote tumor growth, angiogenesis, metastasis and immunosuppression [138-140]. Two distinct subsets of macrophages have been proposed, including classically activated (M1) and alternatively activated (M2) macrophages. Evidently, the infiltrated macrophages in most tumors are M2 phenotype, which provides an immunosuppressive microenvironment for tumor progression [138]. Jonathan et al. reported that targeting tumor-infiltrating macrophages decreased the number of tumor-initiating cells, relieved immunosuppression and improved chemotherapeutic responses in pancreatic cancer [139]. Furthermore, several studies have demonstrated the interaction between TAMs and CSCs [138,141-143]. TAMs are closely associated with CSCs in tumor lesion [144]. Yang et al. demonstrated that TAMs regulate murine breast cancer stem cells through macrophage-induced upregulation of Sox2, mediating by a novel paracrine EGFR/Stat3/Sox2 signaling pathway [141]. Recent studies have shown that FOXM1 promotes macrophage migration and recruitment during inflammation and tumor formation [145-147]. Ren and colleagues have elegantly demonstrated that FOXM1 deficiency did not influence the proliferation of macrophages or their monocytic precursors but impaired monocyte recruitment during liver repair [145]. The same research team has further shown that expression of FOXM1 in macrophages is required for pulmonary inflammation, recruitment of macrophages into the tumor site and lung tumor growth [146,147]. Collectively, those findings strongly support the potential role of FOXM1 in TAMs infiltration and recruitment and tumor development and progression.

The interaction between CSCs and their niche is a complicated and bidirectional process. The niche may maintain self-renewal or dedifferentiation of CSCs by producing stemness factors, and CSCs may affect the niche by inducing EMT or other signaling pathways, such as TGF-β. Although direct evidence of a role for FOXM1 in the PCSC niche is lacking, FOXM1 conceivably has a crucial role in PCSCs partially through regulation of PCSC niche-associated signaling pathways.

## Conclusions and future directions

Evidence confirming the concept that the presence of CSCs contributes to the initiation and progression of PDAC continues to mount. As a proliferation-associated transcription factor, FOXM1 plays pivotal roles in the development of PCSCs via cross-talk with several signaling pathways, including HH, Notch, Bmi1, PI3K/AKT, and Wnt, which are responsible for maintenance of stemness. Moreover, FOXM1 is a key promoter of pancreatic carcinogenesis, functioning as an initiator of the early stages of PDAC development via interaction with signaling pathways related to PanIN and PCSCs. A wealth of data from recent molecular mechanistic studies of CSCs has helped us to more deeply comprehend PCSCs, which should give us a better understanding of the mechanisms that govern the initiation and development of pancreatic cancer. Novel therapeutic strategies targeting PCSCs and thus having a positive impact on clinical outcome in PDAC patients can be envisaged because of this improved understanding. However, the molecular mechanisms by which FOXM1 and other signaling pathways regulate PCSCs remain poorly understood. Additionally, mechanisms governing the PCSC niche must be elucidated. Equally important would be to address the pancreatic cancer specific questions like desmoplasia using the available tissue-specific FOXM1 knockout models of FOXM1. Further studies of the cross-talk of FOXM1 with other signaling pathways as well as studies of the CSC niche also would provide valuable insight into pancreatic cancer pathogenesis and lead to more preventive and therapeutic approaches for PDAC.

## Competing interest

The authors declare that they have no competing interests.

## Authors’ contributions

MG, PW, JC, YG and KX wrote and finalized the manuscript. All authors read and approved the final manuscript.
